# Comparison of Respiratory Effects between Dexmedetomidine and Propofol Sedation for Ultrasound-Guided Radiofrequency Ablation of Hepatic Neoplasm: A Randomized Controlled Trial

**DOI:** 10.3390/jcm10143040

**Published:** 2021-07-08

**Authors:** Heejoon Jeong, Doyeon Kim, Duk Kyung Kim, In Sun Chung, Yu Jeong Bang, Keoungah Kim, Myungsuk Kim, Ji Won Choi

**Affiliations:** 1 Samsung Medical Center, Department of Anesthesiology and Pain Medicine, School of Medicine, Sungkyunkwan University, Seoul 06351, Korea; heejoon.jeong@samsung.com (H.J.); doyeon31.kim@samsung.com (D.K.); dk68.kim@samsung.com (D.K.K.); insun80.chung@samsung.com (I.S.C.); gag_reflex.bang@samsung.com (Y.J.B.); myungsuk91.kim@samsung.com (M.K.); 2 Department of Anesthesiology, School of Dentistry, Dankook University, Cheonan 31116, Korea; keoungah.kim@gmail.com

**Keywords:** dexmedetomidine, hepatocellular carcinoma, percutaneous radiofrequency ablation, propofol, respiration

## Abstract

Patient’s cooperation and respiration is necessary in percutaneous radiofrequency ablation (RFA) for hepatocellular carcinoma (HCC). We compared the respiratory patterns of dexmedetomidine and propofol sedation during this procedure. Participants were randomly allocated into two groups: the continuous infusions of dexmedetomidine-remifentanil (DR group) or the propofol-remifentanil (PR group). We measured the tidal volume for each patient’s respiration during one-minute intervals at five points and compared the standard deviation of the tidal volumes (SDvt) between the groups. Sixty-two patients completed the study. SDvt at 10 min was not different between the groups (DR group, 108.58 vs. PR group, 149.06, *p* = 0.451). However, SDvt and end-tidal carbon dioxide (EtCO_2_) level of PR group were significantly increased over time compared to DR group (*p* = 0.004, *p* = 0.021; ß = 0.14, ß = −0.91, respectively). Heart rate was significantly decreased during sedation in DR group (*p* < 0.001, ß = −2.32). Radiologist satisfaction was significantly higher, and the incidence of apnea was lower in DR group (*p* = 0.010, *p* = 0.009, respectively). Compared with propofol-remifentanil, sedation using dexmedetomidine-remifentanil provided a lower increase of the standard deviation of tidal volume and EtCO_2_, and also showed less apnea during RFA of HCC.

## 1. Introduction

Percutaneous radiofrequency ablation (RFA) is widely performed as a minimally invasive treatment for hepatocellular carcinoma (HCC) due to its relative safety, low risk of complications, and applicability, when compared with surgical resection [1,2]. RFA has been increasingly used as an alternative treatment for unresectable HCC, which often occurs in hepatic reserve-impaired cirrhotic liver cases [3].

Precise placement of the RF electrodes is essential for achieving a good therapeutic response to percutaneous RFA. If the tumor is located on the dome of the liver, regular and shallow breathing of patients is very important for successful treatment [4]. Therefore, despite some discomfort or pain, the patient must control their breathing at the request of the operator during mapping and breathe evenly without body movement during ablation. However, patients who receive RFA usually complain of severe pain during the procedure and sedative drugs may alter the patient’s respiratory pattern. Therefore, adequate levels of sedation and pain control are required during the procedure.

A combination of propofol with remifentanil or fentanyl is often used for the sedation in hepatic RFA. Propofol is associated with a fast onset, short half-life and rapid recovery. However, it has been associated with serious problems such as respiratory depression, and even apnea [5]. Opioids can also cause slow and exaggerated deep breathing. Dexmedetomidine is a highly selective α_2_ agonist with sedative and analgesic effects, which causes a minimal respiratory depression at a clinically effective dose. Therefore, it provides an arousable sedation with minimal changes in the patient’s respiration [6].

In this study, we compared the respiratory patterns of patients in a dexmedetomidine-remifentanil (DR group) or propofol-remifentanil (PR group) sedation during RFA of HCC. The primary outcome was a standard deviation of the tidal volumes (SDvt) during one-minute intervals at 10 min after beginning RFA. We also investigated changes in hemodynamic variables, pain score, interventionist satisfaction, and the incidence of adverse events.

## 2. Materials and Methods

This prospective, randomized controlled trial was approved by the Institutional Review Board (IRB) of the Samsung Medical Center, Seoul, Republic of Korea (IRB No. SMC 2015-06-096-003). This study was registered with the Clinical Research Information Service (http://cris.nih.go.kr; registration No. KCT0003229, accessed on 28 September 2018). Before enrollment, informed written consent was obtained from each patient who received percutaneous RFA in a tertiary care academic center from August 2015 to March 2016. Patients 20–70 years of age with an American Society of Anesthesiologist (ASA) physical status II or III undergoing elective percutaneous RFA under monitored anesthesia care (MAC) to treat single hepatic tumors were included. Exclusion criteria were: sinus bradycardia (heart rate [HR] < 50 bpm/min), heart block greater than the first degree, arrhythmia, severe pulmonary or cardiac disease, cerebrovascular disease, end stage renal disease, and allergy to dexmedetomidine or propofol.

The patients were randomly assigned to the dexmedetomidine-remifentanil (DR) or propofol-remifentanil (PR) group using the computerized random number generator program, www.randomizer.org (accessed on 30 July 2015). An enclosed assignment in a sequentially numbered, opaque, and sealed envelope was allocated to each patient. On the morning of the intervention, this envelope was opened by one author (H.J.) in a blinded manner, and the card inside determined the patient’s group allocation. The author (H.J.) then prepared either propofol or dexmedetomidine for continuous infusion. MAC was performed by two anesthesiologists (J.W.C. and I.S.C.) according to the assigned drug. None of the other anesthesiologists or interventionists involved in patient management or data collection were aware of the group assignment. Patients were also blinded to the group allocation.

None of the patients received premedication. Noninvasive blood pressure, electrocardiography, peripheral oxygen saturation (SpO_2_), HR, and respiratory rate (RR) were monitored continuously throughout the procedure. Vital signs, the Modified Observer’s Assessment of Alertness/Sedation scale (MOAA/S) score, end-tidal carbon dioxide (EtCO_2_) level, the tidal volume for each patient during one-minute intervals, and an infusion dose of each drug were recorded as follows: before sedation (baseline), during ultrasound scanning (ultrasound scan), and then at 5, 10, and 15 min after the RFA was started. Tidal volume was measured using a ventilator circuit with a transparent facial anesthesia mask (0562F, Westmed Inc., Tucson, AZ, USA) that was tightly fitted by a mask harness to prevent breathing leaks. The EtCO_2_ level and RR were measured using gas sample lines connected to the mask and collected with capnography. Six L/min of oxygen was given to the patient through a ventilator circuit. The patient breathed comfortably wearing a facial anesthesia mask that was connected to the ventilator (Carestation 620, GE healthcare, Chicago, IL, USA) and breathing circuit (Moohan Ltd., Gwangju, Korea). The anesthesiologist recorded each tidal volume for one minute at five points (values of tidal volume corresponding to each respiration during the one minute were recorded).

For the DR group, a bolus of 0.1 μg/kg dexmedetomidine (Precedex, Hospira, Inc., Lake Forest, IL, USA) was injected intravenously 3–5 times as a loading dose during the ultrasound scanning. A continuous infusion dose of 0.2–1.0 μg/kg/hr was given during the procedure. For the PR group, a continuous infusion of propofol (Fresofol MCT, Fresenius Kabi, Austria) at a rate of 10–50 μg/kg/min was given during the procedure, and a bolus dose of 0.3 mg/kg was injected just before the start of RFA. The level of sedation in both groups was targeted to a score of 3–4 on MOAA/S during the entire procedure. In both groups, continuous infusion of remifentanil using a target-controlled infusion (TCI) pump (Orchestra^®^, Fresenius Kabi, Brezins, France) was simultaneously performed during the infusion of dexmedetomidine or propofol. The administration of remifentanil was started at a concentration of 1.0 ng/mL, and then adjusted up to 3–5 ng/mL in most cases using a stepwise manner of 0.2 ng/mL based on the patient’s vital signs or any complaints about pain. We defined hypoxia as SpO_2_ < 90%. Apnea was defined as not breathing spontaneously for at least 20 s. We managed adverse respiratory events with a jaw thrust, mask ventilation, or by increasing oxygen flow. Ephedrine, atropine, or nicardipine was administered for adverse hemodynamic events. All anesthetic drugs were discontinued immediately after RFA.

After the procedure, both the interventionist’s and patient’s satisfaction during the procedure were assessed according to a 5-point scale (0: worst, 4: best) score. The radiologists were blinded to patients’ assignments and their satisfaction was obtained in writing by the blinded clinician. Another investigator (D.K.) evaluated the pain score of the patient using the visual analog scale (VAS; 0 = no pain, 10 = worst pain imaginable) and incidence of nausea/vomiting at the post-anesthesia care unit. Sedation time, ablation time, total dose of remifentanil, patient movement (none, mild, or gross), and adverse events (desaturation, apnea, bradycardia or nausea/vomiting) during the procedure were also recorded.

Percutaneous RFA was performed by one of six interventional radiologists under ultrasound (LOGIQ E9; GE Healthcare, Chicago, IL, USA) guidance using fusion imaging (Volume navigation, GE Healthcare) of real-time ultrasound and pre-acquired computed tomography/magnetic resonance images. Contrast (Sonazoid; GE Healthcare, Chicago, IL, USA)-enhanced ultrasound was applied when the lesion conspicuity was not sufficient for accurate applicator placement under fusion imaging guidance. Artificial ascites or pleural effusion were introduced whenever it needed to enhance sonographic window or avoid collateral thermal damage. VIVA RFA (STARmed, Gyeonggi, Korea) or Jet-tip RFA (RF Medical, Seoul, Korea) were used according to the operator’s preference. We aimed to create at least a 0.5 cm ablative margin around the index tumor. Therefore, RFA was continued after repositioning the RF electrode when needed. After the procedure, the needle tract was cauterized to avoid tract bleeding or tumor seeding.

The primary outcome of this study was an SDvt for one-minute intervals at 10 min after the start of RFA, compared between the two groups. The number of patients was calculated based on a pilot study conducted in 14 patients. In the pilot study, the mean ± standard deviation (SD) of the SDvt of the DR and PR groups were 93.89 ± 60.91 and 145.95 ± 78.09, respectively. Thirty patients in each group were required to achieve a power of 80% to detect differences and a significance level of 0.05. Assuming a 10% of dropout rate, we planned to enroll at least 66 patients (33 individuals for each group). Sample size calculation was analyzed by a t-test and performed using nQuery + nTerim 3.0.

Categorical variables were presented as a number and percentage, and continuous variables were expressed as the mean with standard deviation or median with interquartile ranges. Fisher’s exact test was used to compare categorical variables and the Wilcoxon rank sum test was used to determine the significant differences in continuous variables between the two groups. We compared the SDvt measured at each time point using the Wilcoxon rank sum test, and *p*-values were corrected by Bonferroni’s method. We also investigated the change of outcome variables in each group including SDvt, vital signs, and sedation level over time using a paired t-test or a Wilcoxon signed rank test, as appropriate. Finally, we applied a Generalized Estimating Equation (GEE) model to compare the outcome variable trends between the two groups. Statistical analyses were performed using SAS (version 9.4, SAS Institute, Inc., Cary, NC, USA). For all comparisons, a *p*-value < 0.05 was considered to be statistically significant.

## 3. Results

**Figure 1:**
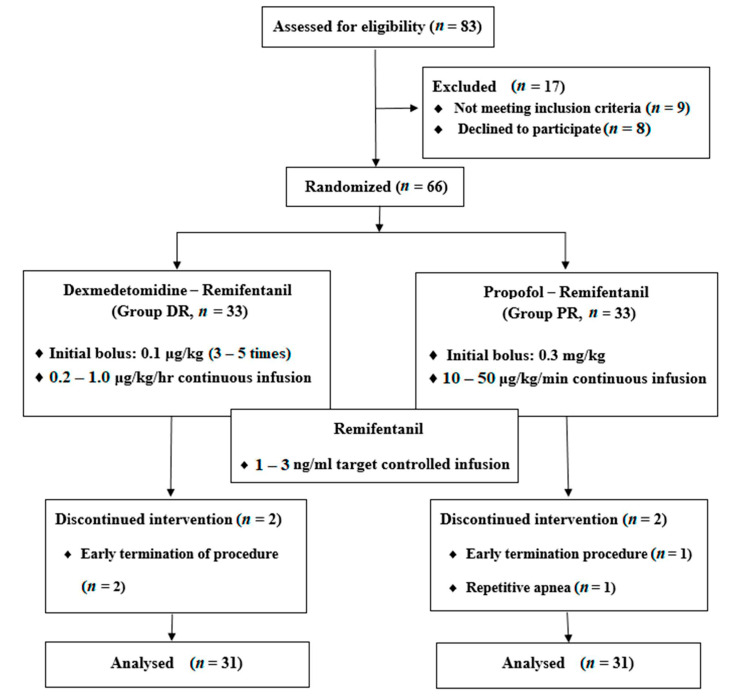
CONSORT flow diagram showing the study protocol and the patients’ progress through the study phases.

Eighty-three patients were eligible for enrollment, but nine of the patients did not meet the inclusion criteria and eight patients declined to participate. The remaining 66 patients were allocated randomly to receive either dexmedetomidine-remifentanil (DR group, *n* = 33) or propofol-remifentanil (PR group, *n* = 33) group. Among them, four patients (two in the DR group; two in the PR group) were excluded because of early termination of the procedure in three cases and repetitive apnea in one case. Thus, the final analysis was performed on 62 patients (Figure 1).

Patient characteristics and operative data are shown in Table 1. There were no significant differences in demographic or technical details during RFA. Sedation and procedure-related outcomes are shown in Table 2.

**Figure 2:**
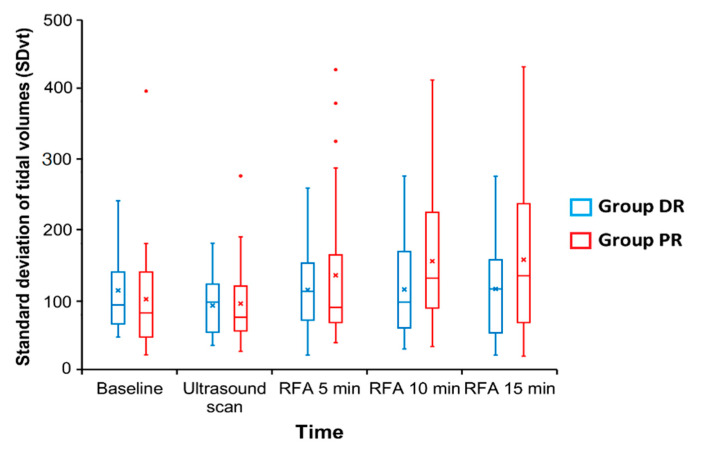
Standard deviation of the tidal volume during sedation between the DR and PR groups. DR, dexmedetomidine-remifentanil; PR, propofol-remifentanil; RFA, radiofrequency ablation.

**Figure 3:**
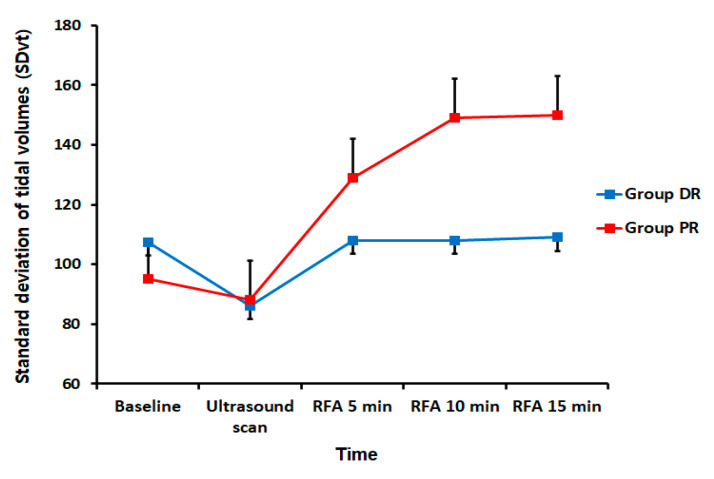
Standard deviation of the tidal volume (SDvt) over time during sedation between the DR and PR groups. The SDvt of PR group was increased over time (*p* = 0.004, ß = 0.142). DR, dexmedetomidine-remifentanil; PR, propofol-remifentanil; RFA, radiofrequency ablation.

The primary outcome, which was the SDvt during the one-minute intervals at 10 min between the two groups, was not statistically different between the two groups (DR group, 108.58 ± 68.54 vs. PR group, 149.06 ± 92.52, *p* = 0.451; Figure 2). However, the trend of SDvt over time was significantly different between the DR and PR groups (*p* = 0.015, ß = −0.151; Figure 3) using the GEE model to compare the trend of outcomes as continuous variables. In other words, the SDvt of the PR group increased over time (*p* = 0.004, ß = 0.142), meanwhile, the SDvt of the DR group was maintained without a significant change during sedation (*p* = 0.810, ß = −0.009). 

**Figure 4:**
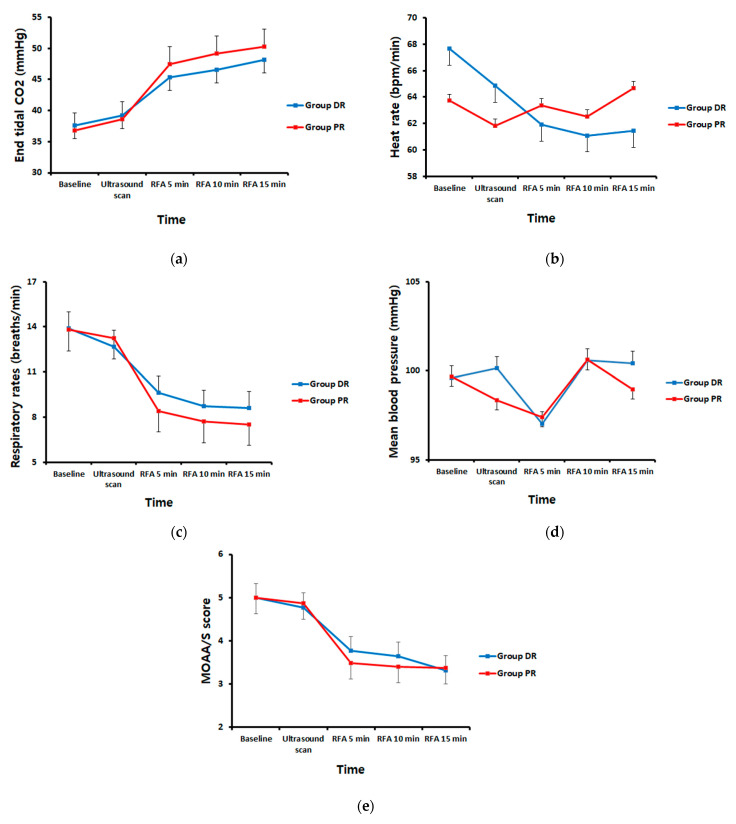
The trends over time of (**a**) end-tidal carbon dioxide (EtCO_2_), (**b**) heart rate (HR), (**c**) respiratory rate (RR), (**d**) mean blood pressure (MBP), and (**e**) modified observer’s assessment of alertness/sedation scale (MOAA/S) score during sedation. The extent in increase of the EtCO_2_ level in the PR group was larger than that of the DR group over time (DR group, *p* < 0.001 and ß = 2.860 vs. PR group, *p* < 0.001 and ß = 3.780). The HR decreased over time in the DR group (*p* = 0.002, ß = −1.650), and showed no significant changes over time in the PR group (*p* = 0.139, ß = 0.670). In both groups, RR decreased over time (*p* = 0.231 ß = 0.350). There was no significant difference in the MBP of the two groups over time (*p* = 0.906, ß = 0.12). The MOAA/S score was comparable over time (*p* = 0.649, ß = 0.02). DR, dexmedetomidine-remifentanil; PR, propofol-remifentanil; RFA, radiofrequency ablation.

Additionally, the trend for the EtCO_2_ level over time was significantly different between the two groups (*p* = 0.021, ß = −0.910). The extent in increase of the EtCO_2_ level in the PR group was larger than that in the DR group over time (DR group, *p* < 0.001 and ß = 2.860 vs. PR group, *p* < 0.001 and ß = 3.780; Figure 4a). The HR decreased over time in the DR group (*p* = 0.002, ß = −1.650; Figure 4b), and showed no significant changes over time in the PR group (*p* = 0.139, ß = 0.670; Figure 4b). In both groups, the RR decreased over time (*p* = 0.231, ß = 0.350), but the trend for the RR was not statistically different between the groups (DR group, *p* < 0.001 and ß = −1.470 vs. PR group, *p* < 0.001 and ß = −1.830; Figure 4c). There was no significant difference in the mean blood pressure (MBP) of the two groups over time (*p* = 0.906, ß = 0.12; Figure 4d). The sedation level, which was measured using MOAA/S was comparable over time and was maintained between 3 or 4 during the procedure in both groups (*p* = 0.649, ß = 0.02; Figure 4e).

The satisfaction level of the radiologists was significantly higher, and the incidence of apnea was lower in the DR group (3.5 ± 0.7 vs. 2.9 ± 0.9, *p* = 0.010; 7 [23%] vs. 18 [58%], *p* = 0.009, respectively; Table 2 and Table 3). There were no serious complications during this study.

## 4. Discussion

In this study, the SDvt during one-minute intervals at 10 min after starting ablation between the DR group and the PR group sedation was not statistically different during RFA of HCC. However, we found that the DR group maintained the SDvt over time without a significant change during sedation. Additionally, the DR group showed less carbon dioxide (CO_2_) retention over time, a lower incidence of apnea, higher associated interventionist satisfaction, and a decrease of HR when compared with the PR group during sedation.

Although ablation therapy of hepatic neoplasm is less invasive and safer than surgery, considerable levels of discomfort and pain can occur in the delivery of radiofrequency energy [7]. Generally, the ideal patient is adequately sedated, breathes regularly, and cooperates with the procedure. Therefore, spontaneous, regular respiration and cooperation with the physician’s requests are ideal for patients during RFA [7]. During this process, the patients must control their breathing according to the request of the operator during the mapping, and breathe evenly without body movement during ablation. Unexpected movement, deep or irregular breathing, or snoring during the procedure may interfere with precise mapping and then lead to a needle displacement during ablation [8]. As a result, treatment of the tumor may be incomplete or insufficient. Therefore, a minimal to moderate degree of sedation and adequate analgesia are needed for a successful procedure.

Propofol has been widely used for various procedures in non-operating room anesthesia because it has a fast onset and shorter recovery time [9]. However, it has the potential to cause muscle relaxation, dose-dependent respiratory depression, and decrease respiratory drive; additionally, aspiration pneumonia has been reported to occur with an incidence of 2.3% following endoscopic submucosal dissection [10,11]. Moreover, it is difficult to control the sedation depth with propofol [12]. Dexmedetomidine is a highly selective α_2_ adrenergic receptor agonist that has analgesic and sedative properties with little effect on the patient’s ventilation [6]. Therefore, we postulated that this characteristic of dexmedetomidine would be more suitable for sedation of patients undergoing RFA of hepatic neoplasms. Many studies have reported that dexmedetomidine did not cause an associated respiratory impairment [13,14]. Mahmoud et al. [6] reported that dexmedetomidine provides sedation, which parallels natural sleep without significant respiratory depression, because dexmedetomidine has the ability to maintain spontaneous ventilation and airway muscle tone. Our study also suggested that dexmedetomidine did not cause either a severe respiratory depression or a significant hemodynamic instability.

Although the SDvt during one-minute intervals at 10 min after the start of ablation was not statistically different between the groups, the SDvt of the DR group was maintained over time without a significant change during the sedation. This indicates that dexmedetomidine has little effect on the breathing pattern during sedation. This is also supported by the finding that the increase of EtCO_2_ levels in the PR group was larger than the DR group over time. The RR trend decreased during sedation and was not statistically different between the groups. Therefore, based on these results we hypothesized that maintaining the patient’s tidal volume with regular breathing is important for effective ventilation.

In another study that compared dexmedetomidine and propofol sedation in RFA for hepatic neoplasms, propofol was associated with a significant reduction of RR in comparison with dexmedetomidine during the procedure, and a greater increase in the partial pressure of arterial carbon dioxide (PaCO_2_) between pre- and post-procedure values, compared to dexmedetomidine [7]. These findings might be associated with the effect of propofol on CO_2_ retention during sedation. CO_2_ retention is usually an important indicator of hypoventilation. Although hypoxia is a more serious problem than hypercapnia in clinical situations, hypoventilation and CO_2_ retention could lead to respiratory depression even in normal oxygen readings [15]. Therefore, as described earlier, the best benefit of using dexmedetomidine for sedation during RFA is its small effect on ventilation.

However, dexmedetomidine requires a loading dose of 1 μg/kg for 10 min for adults to start sedation. In the current study, to compensate for the loading dose delay, a bolus of 0.1 μg/kg dexmedetomidine was injected intravenously 3–5 times during the ultrasound scanning. Additionally, because dexmedetomidine clearance is reduced in patients with hepatic impairment, a dose reduction is required for these patients. Through the protocol described above, it was possible to maintain patients’ respiration and hemodynamic stability, so that the plasma concentration increased slowly during sedation which otherwise may not have reached a sufficient level for analgesia [16]. In this study, both remifentanil consumption and the pain score for patients were comparable between the two groups.

We measured the EtCO_2_ level and RR via a side stream capnography. Generally, the EtCO_2_ values obtained by capnography was correlated well with PaCO_2_ [17,18]. However, in some situations, such as patient noncompliance with nasal cannulas, cannula dislodgement, hemodynamic instability, or when the PaCO_2_ exceeded 60 mmHg, the EtCO_2_ values may not accurately reflect the PaCO_2_ [19,20]. Therefore, we used a mask harness to fit the facial anesthesia mask tightly, which prevented the leaking during respiration. As the characteristics of dexmedetomidine demonstrated less CO_2_ retention without significant changes in SDvt during sedation, dexmedetomidine is thought to be very suitable for procedural sedation for maintaining spontaneous ventilation, especially for procedures that are performed outside the operating room.

There were several limitations to this study. First, we did not confirm the CO_2_ retention using arterial blood gas analysis (ABGA). As we were concerned about the patient’s discomfort or potentially delaying the procedure, and due to the limitations of non-operating room anesthesia, we could not test the ABGA. However, in patients that did not have significant cardiopulmonary dysfunction, the PaCO_2_ can be estimated by using EtCO_2_ value [21]. We also analyzed the values of EtCO_2_ as continuous variables; therefore, the EtCO_2_ trend was monitored during sedation. Second, the anesthesiologist was not blinded to the dexmedetomidine and propofol infusions, because of the differences in the nature of each drug, including delivery system and issues with pain on intravenous injections. However, both the patients and interventionists were blinded, and the sedation was given with an objective protocol using that MOAA/S, which is a reliable test [22]. Third, we could not exclude the effect of remifentanil on the ventilation. We applied a dose of remifentanil to both groups for analgesia. If the patient complained of pain or if the MBP increased during the procedure, we adjusted and administered a dose of remifentanil in both the DR and PR groups. Opioids can induce dose-dependent respiratory depression by directly acting on the respiratory centers in the brainstem [23,24]. The RR is usually slower following opioid overdose [25]. However, remifentanil consumption during RFA was comparable between the groups. Fourth, although we started the infusion of sedatives at the same time, the property of dexmedetomidine with the relatively slow onset compared to propofol could affect the levels of sedation and degree of the SDvt. It might also benefit regulation because it gives clinicians more time to gauge patient individual drug sensitivity.

## 5. Conclusions

In conclusion, dexmedetomidine with remifentanil showed less effect on tidal volume and EtCO_2_ levels compared with propofol with remifentanil during sedation for RFA for HCC. Based on these results, for a smooth and effective procedure, we recommend dexmedetomidine during sedation for RFA of hepatic neoplasm over propofol.

## Figures and Tables

**Table 1:** Characteristics of patients and operative data.

Variables	Group DR (*n* = 31)	Group PR (*n* = 31)	*p* Value
Sex, male/female	21/10	17/14	0.435
Age, years	60.94 ± 10.71	61.94 ± 12.07	0.406
Height, m	1.64 ± 0.09	1.61 ± 0.10	0.291
Weight, kg	67.36 ± 15.27	61.74 ± 11.26	0.080
Body mass index, kg/m^2^	24.84 ± 4.24	23.79 ± 2.90	0.285
ASA physical status, II/III	30/1	27/4	0.354
Snoring history	13 (42)	11 (36)	0.795
Cause of tumor			0.388
Hepatitis B	25 (81)	19 (61)	
Hepatitis C	2 (7)	5 (16)	
Metastasis	3 (10)	4 (13)	
Others	1 (3)	3 (10)	
Child-Pugh classification, A/B	31/0	29/2	0.492
Platelet count, 10^3^/μL	131.68 ± 88.04	140.03 ± 87.29	0.526
Previous treatment ^a^			0.173
Liver surgery	6	8	
RFA	18	13	
TACE	13	17	
Tumor size, mm	17.03 ± 7.76	14.45 ± 5.89	0.193
Tumor location			0.320
Liver surface	7 (23)	3 (10)	
Liver parenchyma	21 (68)	23 (74)	
Vascular structures	3 (10)	3 (10)	
Parietal peritoneum	0 (0)	2 (6)	
Distance to diaphragm, mm	30.19 ± 24.06	28.95 ± 22.15	0.855
Operator, 1/2/3/4/5/6	23/2/4/1/0/1	22/3/1/1/2/2	0.655
Anesthesiologist, 1/2	16/15	14/17	0.799

Values are presented as mean ± standard deviation or number (%). DR, dexmedetomidine-remifentanil; PR, propofol-remifentanil; ASA, American Society of Anesthesiologist; RFA, radiofrequency ablation; TACE, transarterial chemoembolization. ^a^ Multiple responses.

**Table 2:** Sedation and RFA related outcomes.

Outcome	Group DR (*n* = 31)	Group PR (*n* = 31)	*p* Value
Sedation time, min	56.06 ± 27.19	52.00 ± 14.64	0.827
Ablation time, min	22.18 ± 10.41	20.50 ± 6.82	0.989
Total dose of remifentanil, μg/kg	3.82 ± 1.63	3.58 ± 1.42	0.564
Ce of remifentanil, ng/mL			
At RFA 5 min	1.8 ± 0.5	1.9 ± 0.4	0.575
At RFA 10 min	2.0 ± 0.7	2.1 ± 0.6	0.521
At RFA 15 min	2.1 ± 0.7	2.2 ± 0.7	0.710
Patient movement			0.064
none/mild/gross	22/9/0	14/15/2	
MOAA/S score			
At RFA 5 min	3.77 ± 0.56	3.48 ± 0.77	0.095
At RFA 10 min	3.65 ± 0.66	3.42 ± 0.76	0.218
At RFA 15 min	3.39 ± 0.99	3.42 ± 0.67	0.881
After procedure	4.58 ± 0.62	4.42 ± 0.67	0.311
VAS score at PACU (0–10)	0.87 ± 1.34	1.71 ± 1.88	0.053
Operator satisfaction (0–4) ^a^	3.47 ± 0.66	2.93 ± 0.88	0.010
Patient satisfaction (0–4) ^a^	3.29 ± 0.69	3.29 ± 0.82	0.812

Values are presented as mean ± standard deviation or number. RFA, radiofrequency ablation; DR, dexmedetomidine-remifentanil; PR, propofol-remifentanil; Ce, effect-site concentration; MOAA/S, Modified Observer’s Alertness/Sedation scale; VAS, visual analog scale (0 = no pain, 10 = worst pain imaginable); PACU, post-anesthesia care unit. ^a^ Satisfaction was evaluated with a 5-point scale score (0: worst, 4: best).

**Table 3:** Adverse events during sedation.

Variables	Group DR (*n* = 31)	Group PR (*n* = 31)	*p* Value
Apnea (>20 s)	7 (23)	18 (58)	0.009
Desaturation (SpO_2_ < 90%)	0 (0)	1 (3.2)	1.000
Bradycardia (HR < 45 bpm)	4 (13)	3 (10)	0.255
Nausea or vomiting	2 (7)	2 (7)	1.000

Values are presented as number (%). DR, dexmedetomidine-remifentanil; PR, propofol-remifentanil; SpO_2_, peripheral oxygen saturation; HR, heart rate.

## Data Availability

The data presented in this study are available on request from the corresponding author.
